# A Neuroendocrine Tumor of Unknown Primary Origin: A Case Report and Review of the Literature

**DOI:** 10.7759/cureus.65200

**Published:** 2024-07-23

**Authors:** John Patresan, Harsh Patel, Angelica Singh

**Affiliations:** 1 Department of Internal Medicine, New York-Presbyterian Brooklyn Methodist Hospital, Brooklyn, USA; 2 Department of Hematology and Oncology, New York-Presbyterian Brooklyn Methodist Hospital, Brooklyn, USA

**Keywords:** unknown primary origin, neuroendocrine carcinoma (nec), neuroendocrine cardiac, net, dotate

## Abstract

Neuroendocrine tumors (NETs) are uncommon malignancies that develop from neuroendocrine cells which most commonly occur in the GI tract, lung, and pancreas. Treatment courses for these tumors are largely dictated by the primary origin site, which can present diagnostic and therapeutic challenges in NETs of unknown primary origin. Herein, we present a case of an NET of unknown primary origin with significant liver metastases. Our aim is to highlight the key components of the workup of NETs of unknown primary origin and detail the biochemical, histopathological, and imaging modalities as recommended by current literature. We highlight the importance of a multidisciplinary approach to both diagnosis and treatment of these patients as well as touch upon therapeutic options.

## Introduction

Neuroendocrine tumors (NETs) are malignancies that arise from neuroendocrine cells and typically occur in the gastrointestinal tract; however, they can be present in various other organs such as the lung, pancreas, breast, thyroid, and skin [1]. They have the ability to produce vasoactive substances such as serotonin which can cause symptoms such as bronchospasm, flushing, and diarrhea [2]. These tumors also express somatostatin receptors on cell surfaces which lend a great utility for diagnostic approaches such as positron emission tomography (PET)-DOTATATE imaging and therapeutic targets with somatostatin analogues [3]. In a small subset of cases (13%), the primary origin of the NET is not known [4]. In certain clinical scenarios such as advanced NETs that are poorly differentiated, elucidating the primary origin may not change management. However, well-differentiated NETs can have drastic variations in treatment options based on the primary site. Identifying an occult primary site may allow for surgical resection in addition to systemic therapeutic options [4]. Modern clinical trials are designed to be primary site-specific, further emphasizing the need to identify the primary site. Emerging therapies that incorporate radiolabeled octreotide, such as peptide receptor radionuclide therapy, may be utilized in cases of unknown primary origin. Here, we explore a case of a 54-year-old female who was diagnosed with a grade II well-differentiated NET with the majority of metastatic disease burden in the liver, the primary origin of which remains nebulous, with suspicion of cardiac primary.

## Case presentation

The patient was a 54-year-old African-American female with a past medical history of hypertension and diabetes who presented with intermittent cramp-like right upper quadrant abdominal pain for approximately two weeks, associated with nausea at mealtime. She underwent routine blood work with her primary care physician which revealed elevated liver enzymes, prompting a right upper quadrant abdominal ultrasound which revealed multiple heterogeneous masses within the liver. She denied any fever, chills, night sweats, unintentional weight loss, changes in appetite, or changes in bowel habits. She denied oral contraceptive and hormonal therapy use. Prior surgical history consisted of a hysteroscopic removal of a uterine fibroid. There was no prior age-appropriate cancer screening with a mammogram or colonoscopy. She was a non-smoker throughout her life and denied any alcohol or illicit substance use. Her laboratory evaluation revealed a white blood cell count of 7.29 x 10^3^/uL (reference range: 4500-11000 cells/mm^3^), Hb of 14.2 g/dL (reference range: 12.5-17.5 g/dL) with a mean corpuscular volume of 72.6 fL (reference range: 80-100 fL) and platelet count of 271 x10^3^/uL. Her basic metabolic panel was within normal limits with the exception of corrected calcium of 10.9 mg/dL (reference range: 8.5 to 10.5 mg/dL). Her total bilirubin was 0.4 mg/dL (reference range: 0.3 to 1.0 mg/dL), aspartate aminotransferase of 51 unit/L (reference range: 8 to 33 U/L), alanine transaminase of 74 unit/L (reference range: 4 to 36 U/L), alkaline phosphatase of 223 unit/L (reference range: 44 to 147 IU/L), and lipase of 244 unit/L (reference range: 0-160 U/L). She also had a computed tomography (CT) scan of the abdomen/pelvis with intravenous (IV) contrast, which re-demonstrated multifocal liver lesions, with a dominant mass measuring up to 13.5 cm. Additionally, an enlarged heterogeneous uterus was noted, likely representing uterine fibroids.

**Figure 1:**
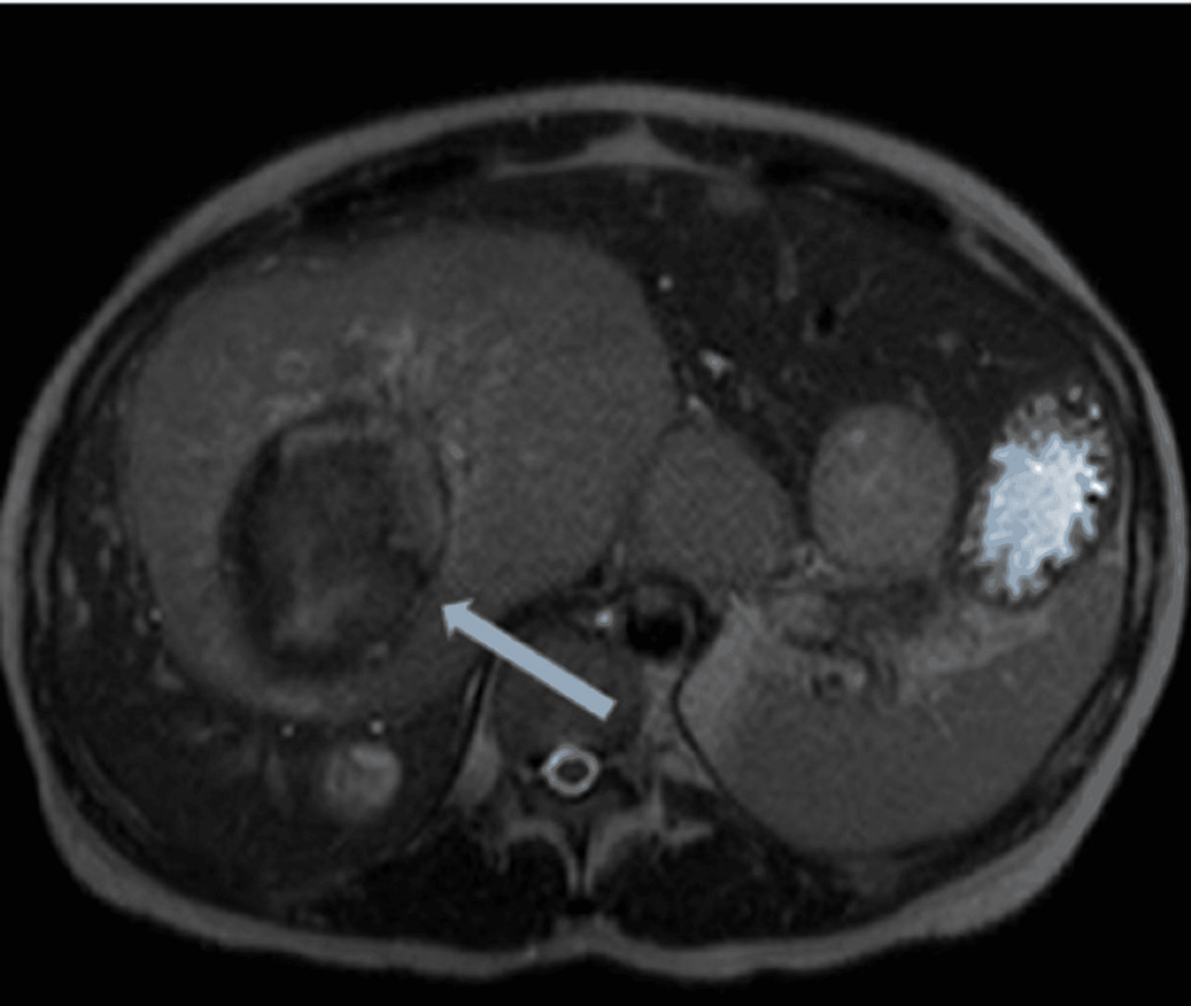
MRI of the Liver With/Without IV Contrast Liver Mass: 10.5 x 13.5 x 13.1 cm of segment 4/8, partially necrotic

Gastroenterology was consulted to evaluate the patient who recommended a triphasic magnetic resonance imaging of the liver in addition to tentative esophagogastroduodenoscopy (EGD) and colonoscopy. Tumor markers were also obtained which revealed an alpha-fetoprotein of 6 ng/mL (reference range: 0 to 40 ng/ml), cancer antigen 19-9 of 18 U/mL (reference range: 19-9: 0-37 U/ml), and a carcinoembryonic antigen of 25.8 ng/mL (reference range: <2.5 ng/ml). She also had a viral hepatitis panel which revealed a positive hepatitis A immunoglobulin G, however, negative immunoglobulin M and a negative hepatitis B and C serology. Triphasic magnetic resonance imaging (MRI) of the liver (Figure 1) revealed multifocal hepatic masses with the largest measuring up to 13.5 cm (right lobe) with areas of necrosis/hemorrhage product. These findings were concerning for metastatic disease. 

**Figure 2:**
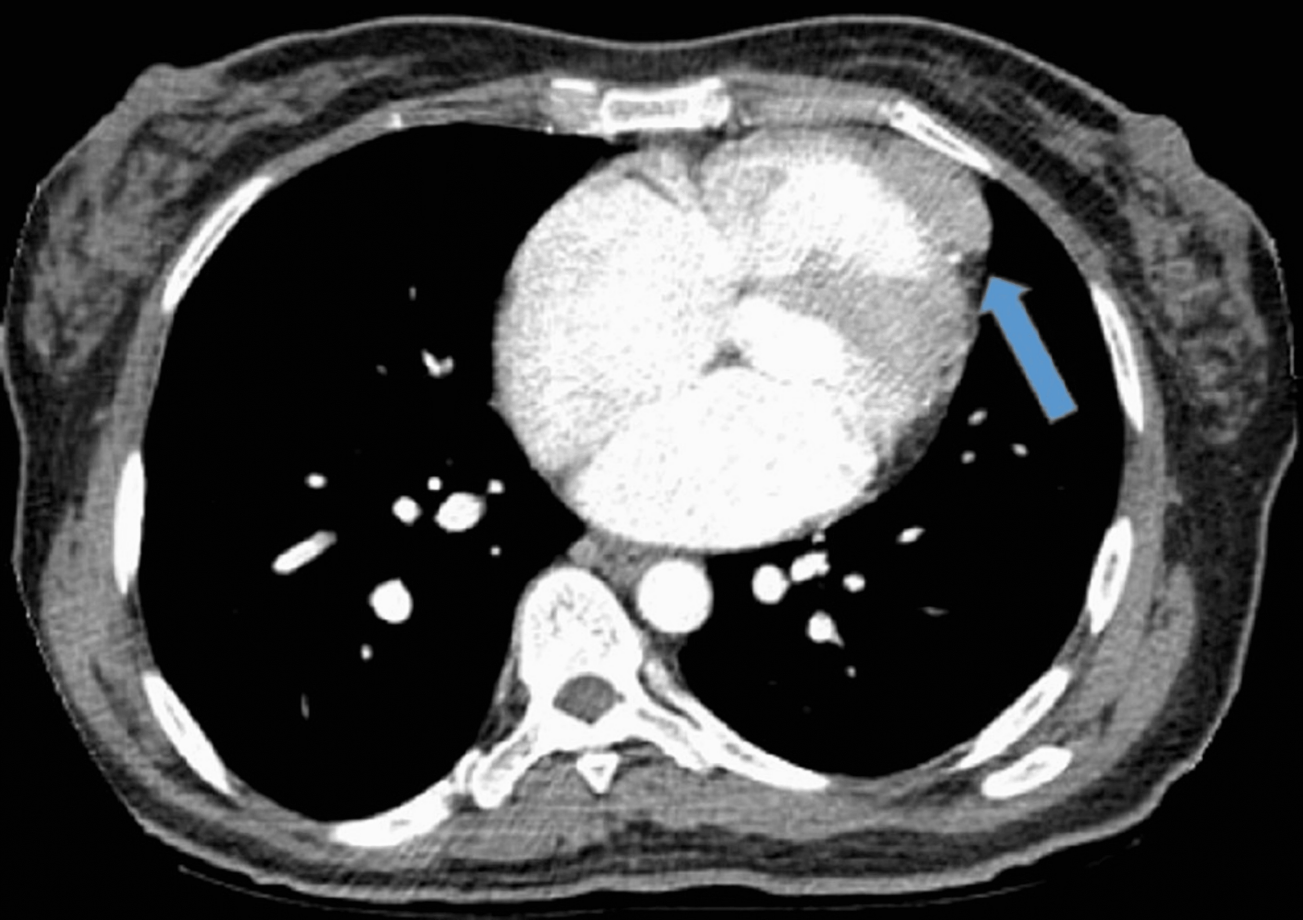
CT Chest With IV Contrast CT chest revealed a 3.6 cm ill-defined mass-like lesion noted in the right ventricle/pericardium; there was no contrast filling ventricular aneurysm, pericardial-based mass/metastasis, or an abnormal lymph node.

The patient then underwent EGD which demonstrated an unremarkable esophagus with evidence of gastritis (pathology of which eventually revealed chronic active H. Pylori-associated gastritis) and normal duodenum. The colonoscopy did not reveal any mass lesions, only diverticula in the sigmoid colon and non-bleeding internal hemorrhoids. Hematology/Oncology was then consulted who recommended an interventional radiology-guided liver biopsy to evaluate further as well as CT chest with IV contrast (Figure 2) to complete the staging work-up. CT chest noted a 3.6 cm ill-defined mass-like lesion, described as a left anterior pericardial soft tissue mass inseparable from the right ventricle. A transthoracic echocardiogram revealed moderately elevated pulmonary artery systolic pressure to 50mmHg as well as increased left ventricular wall thickness and a mildly dilated right ventricle. The ejection fraction was estimated at 60-65% with normal left ventricular systolic function and normal diastolic function. CT chest with IV contrast revealed a 3.6 cm ill-defined mass-like lesion noted in the right ventricle/pericardium; there was no contrast filling ventricular aneurysm, pericardial-based mass/metastasis, or an abnormal lymph node. 

**Figure 3:**
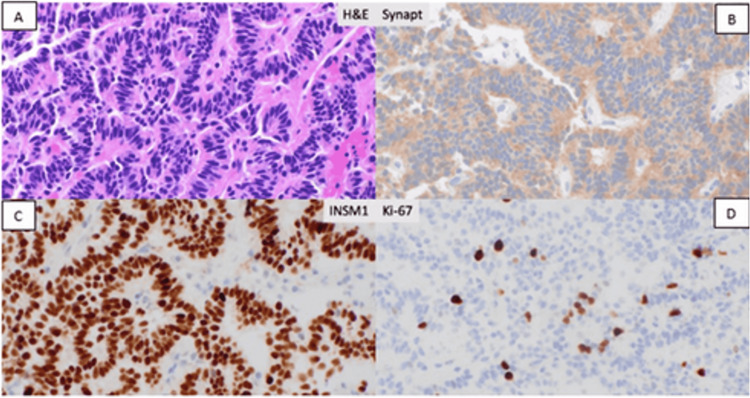
Liver Biopsy With H&E and Immunostaining at 400x Magnification Liver biopsy was performed revealing a well-differentiated neuroendocrine tumor, grade 2 (Ki-67 5-8%). A) H&E; B) Synaptophysin; C) INSM1; D) Ki-67

Liver biopsy (Figure 3) was performed revealing a well-differentiated NET, grade 2 (Ki-67 5-8%). Immunohistochemistry was negative for cytokeratin (CK) 20 and cytokeratin (CK) 7 and suggestive of fairly well-differentiated NET Ki67 5-8%. CK7, CK20, thyroid transcription factor 1 (TTF1), caudal type homeobox 2 (CDX2), GATA3, paired-box gene 8 all negative, with synaptophysin and insulinoma associated 1 (INSM1) positive. There was intact expression of mismatch repair protein (MMRP).

**Figure 4:**
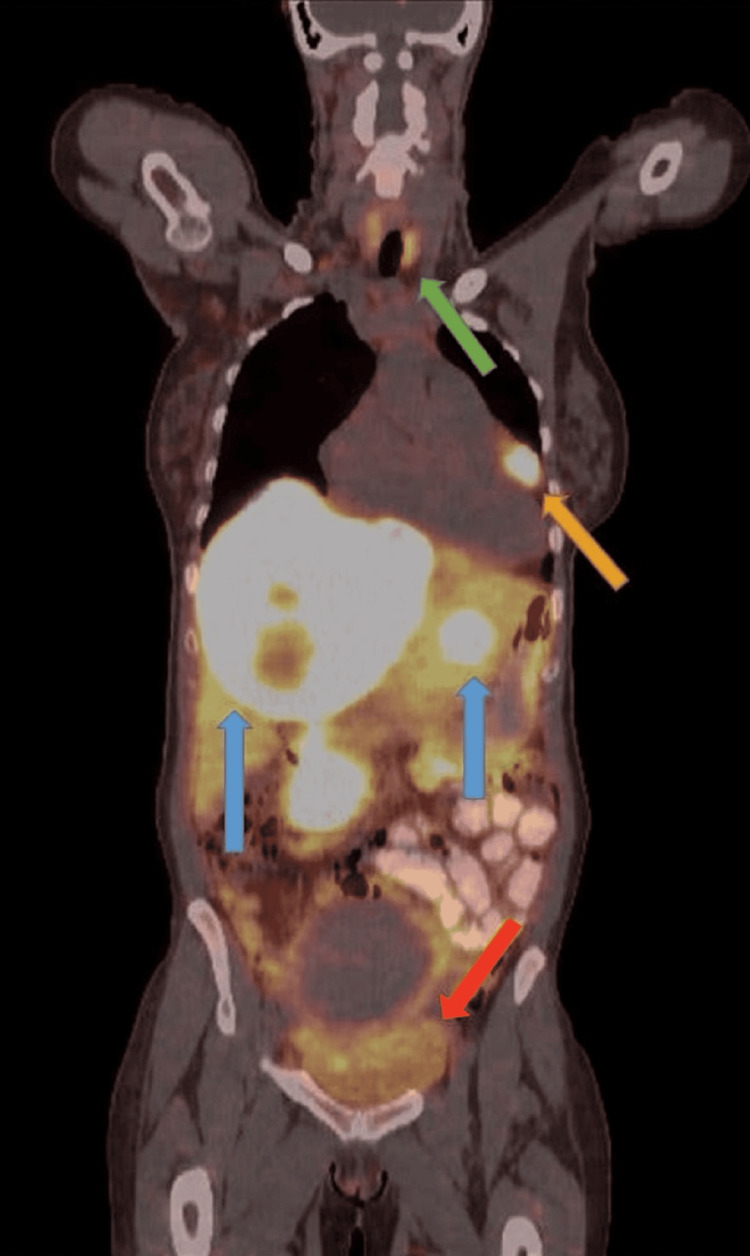
PET DOTATATE There were multiple intensely DOTATATE avid bilobar hepatic masses, including dominant segment 4/8 centrally photopenic/necrotic mass, corresponding to the biopsy-proven neuroendocrine tumor (blue arrow). Also, there was an intensely DOTATATE avid left anterior pericardial soft tissue mass inseparable from the right ventricle, probably nodal versus metastatic involvement (orange arrow). There were additional low-level DOTATATE avid prominent bilateral supraclavicular, mediastinal, and abdominopelvic lymph nodes, with uptake below the background level, possibly reactive versus disease-related (green arrow). There was enlarged leiomyomatous uterus with heterogeneous DOTATATE avidity. Pelvic ultrasound was consistent with a large myoma measuring up to 8 cm (red arrow).

Molecular testing was also obtained and the patient was subsequently discharged with close outpatient follow-up for a PET-DOTATATE scan to determine the extent of disease and identify the primary origin. Of note, the patient denied any carcinoid symptoms such as wheezing, flushing, or diarrhea. She was initiated on monthly lanreotide until PET-DOTATATE could be obtained. Her PET-DOTATATE (Figure 4) revealed multiple intensely DOTATATE avid bilobar hepatic masses, including dominant segment 4/8 centrally photopenic/necrotic mass, corresponding to a biopsy-proven NET. Also, an intensely DOTATATE avid left anterior pericardial soft tissue mass inseparable from the right ventricle, probably nodal versus metastatic involvement. Additional low-level DOTATATE avid prominent bilateral supraclavicular, mediastinal, and abdominopelvic lymph nodes, with uptake below background level, possibly reactive versus disease-related. Enlarged leiomyomatous uterus with heterogeneous DOTATATE avidity. Pelvic ultrasound was consistent with a large myoma measuring up to 8 cm.

The patient was presented at a multidisciplinary tumor board, where locoregional therapy of the liver was discussed. The treatment plan was to start with sublobar right hepatic lobe arterial radioembolization using yttrium 90 (Y90) along with monthly lanreotide injections. The patient is currently undergoing mapping for the arterial chemoembolization procedure.

## Discussion

NETs are uncommon malignancies that develop from neuroendocrine cells and typically occur in the gastrointestinal tract, lung, and pancreas although can develop in various other organs [5]. These malignant cells possess the ability to secret bioactive substances such as serotonin which can manifest with symptoms such as flushing and diarrhea, although non-functioning NETs can also occur [5]. The constellation of “carcinoid-like" symptoms or syndromes such as persistent peptic ulcer disease in the case of gastrinomas, may lead the clinician to discover these cancers; however, this is not always the case. In certain instances, the tumor can present in an ambiguous manner without a clear primary, especially if non-functioning such as in our case. Treatment in such scenarios can prove to be challenging, therefore emphasis is needed on diagnostic tools such as imaging techniques as well as novel molecular signatures histologically and from genomic sequencing for primary site identification [5]. Advancements in such techniques could allow for a more favorable prognosis. Clinically, a patient may present with symptoms related to the anatomical location of the primary or metastasis such as abdominal pain from liver metastasis or dyspnea from a bronchial NET [5]. Biochemical tests can prove to be of value as well. Twenty-four-hour urinary 5-hydroxyindoleacetic acid which is a breakdown product of serotonin is typically elevated more so in small intestinal NETs [4]. Gastrin-producing tumors predominantly arise in the duodenum and pancreas [5]. In the case of functioning NETs, serum measurements of hormones such as fasting gastrin, proinsulin, c-peptide, glucose, glucagon, vasoactive intestinal peptide, somatostatin, and adrenocorticotropic hormone can help elucidate the primary site [5,6]. Immunohistochemical stains have shown to be quite useful in ascertaining the primary source of an NET with certain limitations. CDX2 is a marker shown to be expressed in well-differentiated NETs of intestinal origin; however, it can also be seen in colorectal adenocarcinoma [5]. Insulin gene enhancer protein or paired-box gene 8 expression can suggest pancreatic, duodenal, or rectal origin [5]. TTF-1 is expressed in some poorly differentiated and some well-differentiated bronchial NETs although can be noted in other various extra-pulmonary NETs as well, limiting its specificity [5]. Our case in particular did not express any of the above markers to help suggest the origin of the tumor.

Imaging techniques in the last few decades have demonstrated remarkable efficacy in diagnosing malignancies of unknown primary origin. Beyond initial staging with multiphasic CT or magnetic resonance imaging of the abdomen and pelvis and computerized tomography of the chest, the use of gallium-68 (Ga-68) DOTATATE positron emission tomography has been remarkable in identifying metastatic disease in NETs [5]. There could be utility in using the latter modality in discovering an occult primary of unknown origin. The mechanism of the scan relies on radiolabeled peptides with varying affinities for somatostatin receptors [5]. A study done by Ambrosini et al. found that Ga-68 DOTATATE scans were able to localize 3 out of 4 occult primary neuroendocrine neoplasms [6]. In our case, the Ga-68 PET-DOTATATE revealed intense avidity in the anterior pericardial region involving the right ventricle suggesting the possibility of a primary source. This modality of PET imaging provides superior diagnostic value in detecting cardiac involvement in NETs when compared to conventional morphologic CT imaging [7]. Interestingly, our patient with apparent cardiac involvement did not demonstrate any evidence of carcinoid heart disease including valvular dysfunction or right heart failure. Whether cardiac involvement of NETs (primary or metastatic) and carcinoid heart disease are mutually exclusive or two distinct pathologies is unclear [7]. Cardiac NETs are exceptionally rare and are attributed more so to metastatic disease rather than primary [8]. Metastatic disease should be excluded before a NET of cardiac primary can be diagnosed [8]. It is unlikely for an NET of the heart to present without diffuse metastatic disease [8]. In our case, we do have notable liver involvement. The utilization of PET-DOTATE imaging has been found to increase the ability to detect cardiac lesions in both ileal and non-ileal NETs, which is what ultimately helped us identify the cardiac involvement in our patient [9].

To date, it remains unclear what the primary source of the neuroendocrine malignancy is in this patient despite endoscopic interventions, imaging modalities, and an array of histopathological tests. The multi-focal scattered lesions of the liver suggest metastatic etiology. The avid uptake in the pericardium certainly raises the suspicion of a primary culprit. There does not appear to be any specific immunohistochemical marker utilized for cardiac origin. Cardiac NETs have not been well established on the basis of cytokeratin positivity [10]. Whether other imaging techniques such as cardiac MRI or technetium 99-nuclear scan can be applied to further characterize these masses is a question that needs to be answered. Lastly, treatment in neuroendocrine carcinomas of unknown primary origin has not been well established. In addition to biochemical therapy with lanreotide, locoregional treatment with Y-90 was employed in our patient given the high burden of disease present in the liver. Locoregional therapy appears to be well tolerated in patients with extensive liver metastasis with infrequent adverse toxicities [11]. According to Tsang et al., in a population-based cohort of patients with metastatic liver-dominant NETs, approximately 86% of patients achieved a partial response or stable disease with only 12% demonstrating progression [12]. Further data is needed on whether to pursue a more systemic-based approach or to focus on locoregional therapies for NETs of unknown origin. The standard of care for well-differentiated NETs includes long-acting somatostatin analogues as a first-line approach. Further modalities of systemic therapy as second-line include mTOR inhibitors such as everolimus, interferon-alpha, and tyrosine kinase inhibitors such as sunitinib [13]. In gastroenteropancreatic NETs, patients may benefit from ^177^Lu-DOTATE which is a radioactive isotope that selectively binds to neuroendocrine cancer cells that express the somatostatin receptor and induce apoptosis per the NETTER-1 trial [14]. Lastly, immunotherapy which aims to enhance the ability of the cytotoxic T-cell to help recognize and cause tumor cell death has been explored. Thus far, monotherapy does not appear to be effective although there is some promise of using dual immunotherapy as a tertiary line of treatment upon progression [15]. Failure to identify tumor-specific therapies may contribute to a worse prognosis of NETs with unknown origin as compared to metastatic NETs with a known primary site [16]. 

## Conclusions

In conclusion, the collaborative data obtained from biochemical, histopathological, and imaging modalities are needed not only to identify the origin of primary NETs but also to provide superior treatment modalities for the future. NETs tend to have heterogeneous behavior when it comes to aggressiveness and clinical burden. Identification of the primary can be challenging at times, although knowing the anatomical origin of such can be useful to the clinician, as the tumor may be amenable to locoregional treatment. Thus far, we have utilized the somatostatin expression of these tumors for diagnostic techniques such as the Ga-68 PET-DOTATE and treatment strategies such as peptide receptor radionuclide therapy. In the field of oncology, next-generation sequencing of the tumor and peripheral blood has provided clinicians with relevant molecular alterations that can be targeted for therapy. Hopefully, more research can be done utilizing this technique in the future to help open more doors for therapeutic options.
